# Comparative analysis of drought stress-induced physiological and transcriptional changes of two black sesame cultivars during anthesis

**DOI:** 10.3389/fpls.2023.1117507

**Published:** 2023-02-21

**Authors:** Xiaohui Wang, Min Wang, Gui Yan, Huiyi Yang, Guangwei Wei, Tinghai Shen, Zehua Wan, Wei Zheng, Sheng Fang, Ziming Wu

**Affiliations:** ^1^ Key Laboratory of Crop Physiology, Ecology, and Genetic Breeding, Ministry of Education/College of Agronomy, Jiangxi Agricultural University, Nanchang, China; ^2^ Institute of Garden Science and Technology, Nanchang City Gardening Service Center, Nanchang, China; ^3^ Crop Cultivation Laboratory, Jiangxi Institute of Red Soil and Germplasm Resource, Nanchang, China

**Keywords:** black sesame, drought stress, transcriptome, glutathione and ethylene biosynthesis, antioxidant system, anthesis

## Abstract

Sesame production is severely affected by unexpected drought stress during flowering stage. However, little is known about dynamic drought-responsive mechanisms during anthesis in sesame, and no particular attention was given to black sesame, the most common ingredient in East Asia traditional medicine. Herein, we investigated drought-responsive mechanisms of two contrasting black sesame cultivars (Jinhuangma, JHM, and Poyanghei, PYH) during anthesis. Compared to PYH, JHM plants showed higher tolerance to drought stress through the maintenance of biological membrane properties, high induction of osmoprotectants’ biosynthesis and accumulation, and significant enhancement of the activities of antioxidant enzymes. For instance, the drought stress induced a significant increase in the content of soluble protein (SP), soluble sugar (SS), proline (PRO), glutathione (GSH), as well as the activities of superoxide dismutase (SOD), catalase (CAT), and peroxidase (POD) in leaves and roots of JHM plants compared to PYH plants. RNA sequencing followed by differentially expressed genes (DEGs) analysis revealed that more genes were significantly induced under drought in JHM than in PYH plants. Functional enrichment analyses disclosed that several pathways related to drought stress tolerance, such as photosynthesis, amino acids and fatty acid metabolisms, peroxisome, ascorbate and aldarate metabolism, plant hormone signal transduction, biosynthesis of secondary metabolites, and glutathione metabolism, were highly stimulated in JHM than in PYH plants. Thirty-one (31) key highly induced DEGs, including transcription factors and glutathione reductase and ethylene biosynthetic genes, were identified as potential candidate genes for improving black sesame drought stress tolerance. Our findings show that a strong antioxidant system, biosynthesis and accumulation of osmoprotectants, TFs (mainly ERFs and NACs), and phytohormones are essential for black sesame drought tolerance. Moreover, they provide resources for functional genomic studies toward molecular breeding of drought-tolerant black sesame varieties.

## Introduction

Improving crops’ productivity and quality in the current situation of climate change is challenging. In fact, unexpected abiotic and/or biotic stresses occur during crop plants’ life cycles, causing considerable losses in agriculture production. Among diverse abiotic stresses, numerous studies have shown that drought is the most adverse one, and it causes significant decreases in crop yields and quality traits (Wang et al., 2011; Wang et al., 2019). To mitigate the harmful effects of drought, plants have evolved various physiological and molecular mechanisms, such as induction of diverse family genes and antioxidant defense systems, reinforcement or maintenance of biological membranes’ structure and properties, and accumulation of osmoprotectants (sugar, proteins, proline, and glutathione, GSH) in cells (Chaves et al., 2003; El-Sharkawy, 2004; Najla et al., 2016; Sharma et al., 2017; Mahmood et al., 2020; Jia et al., 2021). Under drought stress conditions, the expression patterns of numerous genes are altered or induced to activate physiological and defense systems (Savoi et al., 2016). The antioxidative mechanisms include enzymatic and non-enzymatic reactions in plant cells and are mediated mainly by peroxidases (POD), catalase (CAT), and superoxide dismutase (SOD) (Impa et al., 2012; Li et al., 2013). It is demonstrated that GSH synthesis and ethylene accumulation improve crop plants’ resistance to drought (Khan et al., 2015). GSH is the predominant reducing thiol in plant cells, and its reduced form plays critical functions in reactive oxygen species (ROS) detoxification (Couto et al., 2016). Phytohormone ethylene regulates several physiological processes, such as growth, flowering, senescence, and stress responses (Ullah et al., 2018).

Sesame belongs to the superficial root plants and is primarily cultivated in tropical and subtropical areas worldwide, where it is exposed to intermittent droughts (Dossa et al., 2016; Arslan et al., 2019; Liang et al., 2021). In 2019, the world’s total harvested area of sesame was around 12.82 Mha, with only about 6.55 Mt of sesame seeds, of which approximately 60% were from Asia (Faisal et al., 2016). Although the sesame plant is tolerant to drought stress compared to other oilseed crops, progressive or prolonged water deprivation significantly affects its growth, development, yield components (reduction of the number of capsules per plant, grains per capsule, and 1000-grain weight), and quality (Bahrami et al., 2012; Dissanayake et al., 2019; Sepideh et al., 2019). Among sesame seeds of different colors, black seeds have higher demand and are priceless, especially in East Asia, where they represent a key ingredient in traditional medicine (Dossa et al., 2018; Wang et al., 2018). They possess various physiological properties, including high antioxidative, anti-nitrosative, anti-obesity, and protective effects against metabolism illness (Panzella et al., 2012; Jin et al., 2014; Ruslan et al., 2018). Accordingly, it is of particular interest to investigate stress-responsive mechanisms in black sesame to enhance its productivity. Unfortunately, litter attention was given exclusively to black sesame response to drought stress. Moreover, albeit some studies were conducted on sesame response to drought stress (Dossa et al., 2017; Dossa et al., 2020; Fang et al., 2022), knowledge of drought-responsive mechanisms in sesame plant is still limited and more candidate genes are likely to be identified.

Anthesis is a developmental stage in the plant life cycle. Studies in many plant species, including maize (Turc and Tardieu, 2018), legumes (Fang et al., 2009), and *Arabidopsis thaliana* (Nevyl and Battaglia, 2021), have shown that water deficit during flowering stages is the most dangerous, causing significantly lost in crop production. Due to climate change occasioning alteration of soils’ physicochemical properties and growing conditions, drought stress of different intensities often accompanies the whole flowering stage of sesame plants. Hence, we need to analyze physiological responses and dynamic transcriptome profiling of black sesame genotypes to drought stress occurring during flowering periods. A deeper investigation and understanding of drought-induced physiological and molecular mechanisms, together with the identification of candidate genes underlying drought tolerance in black sesame is a key step to developing high-yielding and drought-tolerant varieties.

In the present study, we analyzed the drought-responsive mechanisms of two black sesame cultivars widely cultivated and used in China. We examined morphological changes and investigated diverse physiological parameters, including the content of chlorophyll, MDA, soluble sugar, soluble protein, free proline, and glutathione, and the enzymatic activity of SOD, POD, and CAT at different time points of induced drought stress during anthesis both in leaves and roots. Based on the contrasting physiological responses of the two cultivars to the induced drought, we carried out a comparative dynamic transcriptome analysis and revealed DEGs and differently induced pathways. In addition, we examined the expression patterns of glutathione reductase and ethylene biosynthetic genes and identified potential candidate genes for drought tolerance improvement in (black) sesame. The results were further validated through quantitative reverse transcription-polymerase chain reaction (RT-qPCR) analysis. Our findings provide an overview of drought tolerance mechanisms in black sesame and fundamental resources for genomic studies to dissect the regulation network of drought stress in sesame.

## Materials and methods

### Plant materials and drought stress conditions

Two black sesame cultivars, Jinhuangma (JHM) and Poyanghei (PYH), widely cultivated and used in China were assessed in this study. They were provided by the Key Laboratory of Crop Physiology, Ecology, and Genetic Breeding, Ministry of Education, College of Agronomy, Jiangxi Agricultural University (Nanchang, Jiangxi province, China). These varieties were selected based on their cultivation history and performance in fields under various environmental conditions. In fact, our group screened hundreds of sesame plant materials *via* field experiments and selected these two native varieties to clarify the mechanisms underlying black sesame tolerance to drought stress. The cultivar JHM was relatively tolerant to drought, while PYH was sensitive and possessed a high per plant yield in the north of Jiangxi province.

The experimentation was performed in a greenhouse at Jiangxi Agricultural University in 2020. Seeds of the two cultivars were sown float tray until the two true-leave stages. Then, they were transferred into plastic pots (Diameter×Height: 19×27 cm) containing 7.5 kg of soil. To better control water status, the soil water content was measured using a soil moisture probe, 20 cm long (probes inserted vertically into the pots). All the sesame seedlings were watered normally (soil watered daily to 25 ± 5%) until they reached the flowering period (85 days after germination). Thereafter, the drought stress was imposed on other treatments by keeping the soil moisture content at the level of 10 ± 5% for three (T1), five (T2), and seven days (T3), which stand for mild, moderate, and severe drought stress, respectively. Each treatment was composed of twenty individual plants. Middle leaf and root samples from drought stress were sampled at the end of each treatment for physiological analyses. Prior to the induction of the drought stress (the starting day), middle leaves and roots were sampled to constitute control samples (CK) in order to investigate changes in a dynamic manner. Other root samples were prepared for RNA sequence. All the samples were immediately frozen in liquid nitrogen and stored at -80 °C until use. Each sample was analyzed in triplicate.

### Physiological analysis

The chlorophyll content of leaves was determined using the SPAD meter as described previously (Naus et al., 2010; Ling et al., 2011). MDA content was measured *via* the thiobarbituric acid method (Castrejón and Yatsimirsky, 1997). The activity of SOD and CAT was assayed following the method described by García-Triana (García-Triana et al., 2010) and Zhao and Shi (Zhao and Shi, 2009), respectively. POD activity was measured as described in a previous report (Wang et al., 2014). Free proline content was determined based on the spectrophotometric method described by Vieira (Vieira et al., 2010). Soluble sugar content was determined using the anthranone reagent (Bodelón et al., 2010). Finally, soluble protein content was measured by the coomassie brilliant blue G-250 (Lowry et al., 1951).

### RNA sequencing and data assembly

Total RNA from root samples was extracted using the Trizol reagent kit (Invitrogen, Carlsbad, CA, USA) according to the manufacturer’s protocol. RNA quality was assessed on an Agilent 2100 Bioanalyzer and checked using RNase-free agarose gel electrophoresis. Next, each sample RNA was PCR amplified and sequenced using the Illumina Novaseq6000 Sequencing System by Gene Denovo Biotechnology Co. (Guangzhou, China). To get high-quality clean reads, reads were further filtered by fastp (Chen et al., 2018). The mapped reads of each sample were assembled with StringTie v1.3.1 in a reference-based approach (Pertea et al., 2015; Pertea et al., 2016). For each transcript an FPKM value was calculated to quantify its expression abundance and variations using RSEM software (Dewey and Bo, 2011). Principal component analysis (PCA) was performed in R using the “prcomp” package.

### Differentially expressed genes and enrichment analysis

The DESseq2 software (Love et al., 2014) was used to detect DEGs between two different groups with the criteria of false discovery rate (FDR) below 0.05 and absolute fold change ≥ 2. The HISAT2 program (Kim et al., 2015) was used to align the clean reads to the sesame reference genome (“*S_indicum*_v1.0”, https://www.ncbi.nlm.nih.gov/data-hub/taxonomy/4182/) and to obtain information regarding genomic loci and characteristics unique to the sequenced samples. GO (Genes Ontology, http://geneontology.org/), and KEGG (Kyoto Encyclopedia of Genes and Genomes, http://www.genome.jp/kegg/kaas) enrichment analysis for the DEGs were performed using GO seq and KOBAS (2.0) software, respectively. According to the GO annotation result, the DEGs were mapped to GO terms in the Gene Ontology database, and significant enrichment terms were detected at the threshold P-value <0.05. Similarly, KEGG pathways were assigned to the assembled sequences using the online KEGG Automatic annotation server and enrichment analysis.

### RT-qPCR

We isolated total RNA from each root sample and synthesized first-strand cDNAs following the reported methods by Wei et al. (Wei et al., 2019). Real-time quantitative PCR (RT-qPCR) was carried out in CFX96 (BioRad) with the SYBR Green Perfect mix (TaKaRa, Dalian, China). All samples were analyzed in triplicates. Relative expression levels of each gene were computed using the 2^–ΔΔCT^ method (Livak and Schmittgen, 2001). The sesame gene *β-actin* (ncbi_105159390) was used to normalize the genes’ expression levels (Li et al., 2017; Su et al., 2022). The primers were designed with Primer5 and are listed in **Table S1**.

### Data analyses

Statistical analyses of all traits were conducted using SPSS 17.0 software, and the data are presented as the mean ± SD of three replicates. The standard error is shown as an estimate of variability, and Duncan’s multiple test was used to determine statistical differences at *P* < 0.05.

## Results

### Morphological and physiological responses of the two sesame cultivars to drought-induced stress during anthesis

To access the drought-responsive mechanisms of JHM and PYH during anthesis, we investigated morphological and physiological changes after three (T1), five (T2), and seven (T3) days of stress induction. Morphological observations showed that the drought stress caused symptoms of yellowing, drooping, and wilting of leaves of plants of both cultivars (**Figure S1**). However, compared to PYH plants, JHM plants were less affected at T3 (**Figures S1C–F**), indicating they have suffered less damage from drought stress. The yellowing symptom is generally caused by a decrease in chlorophyll content of plants exposed to drought stress (Bhargava and Sawant, 2012; SeyedYahya and Hamideh, 2016; Gurumurthy et al., 2019). Supportively, leaves chlorophyll content analysis revealed a significant decrease in chlorophyll content of the two cultivars (**Figure S2**). For instance, after five days of stress exposure, the leaf chlorophyll content of JHM and PYH plants exhibited a decrease of 23.75% (from 39.87 to 30.40 SPAD) and 30.78% (from 41.80 to 28.93 SPAD), respectively.

**Figure 1:**
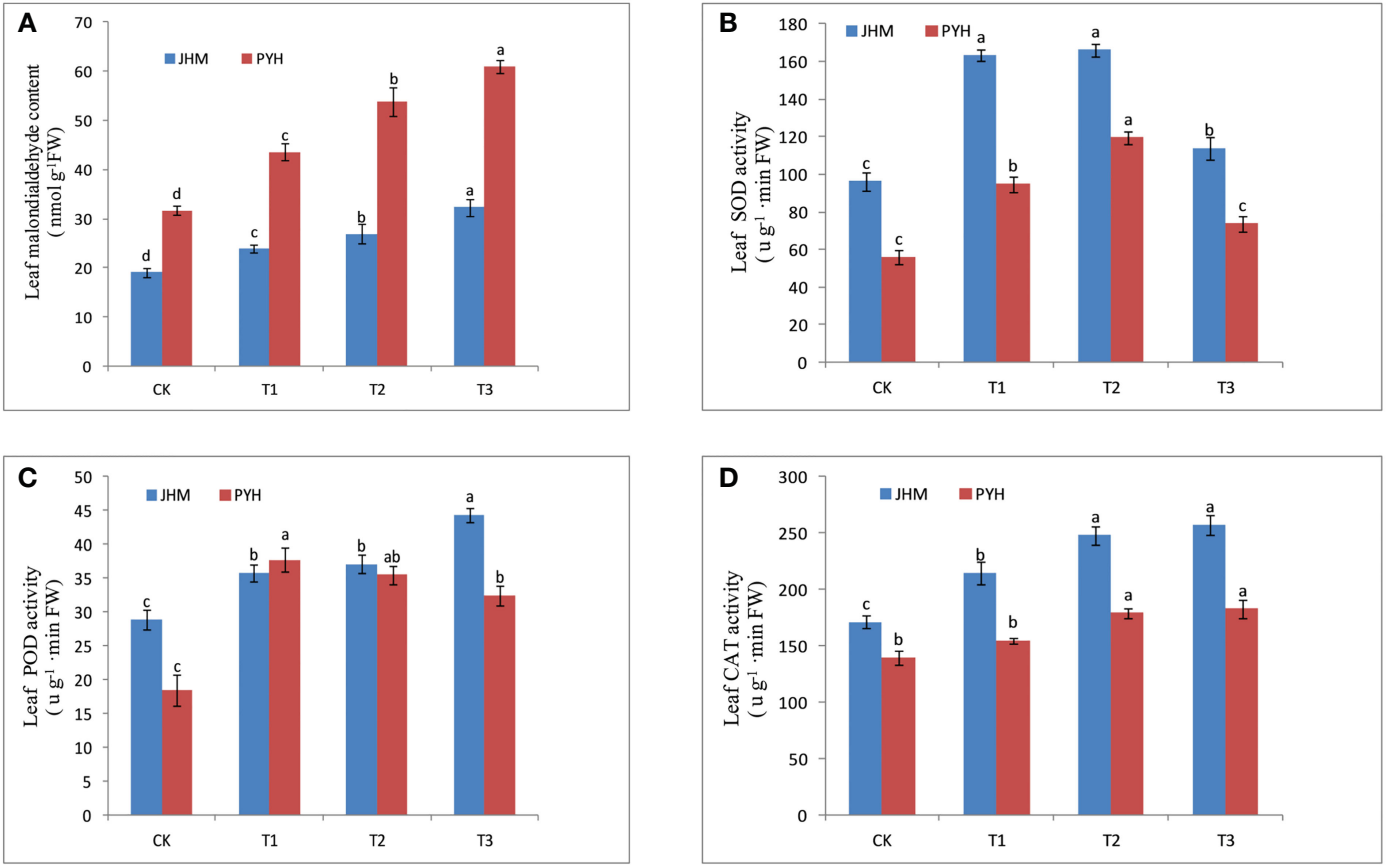
Antioxidation status in leaves of JHM and PYH plants under drought stress during anthesis. **(A)** Malondialdehyde content; **(B)** Superoxide dismutase activity; **(C)** Peroxidase activity; **(D)** Catalase activity. CK, T1, T2, and T3 indicate plants were stressed for 0, 3, 5, and 7 days, respectively. The different lowercase letters indicate significant differences at the P<0.05 probability level.

As drought stress promotes the synthesis of oxidants, we analyzed the malondialdehyde (MDA) content and the activity of antioxidative enzymes superoxide dismutase (SOD), peroxidase (POD), and catalase (CAT) in leaves and roots of the two cultivars at the different time points (**Figures 1**, **S3**). The MDA content of the leaves and roots of the two cultivars was significantly increased along with the drought stress duration (**Figures 1A**, **S3A**). Compared to JHM, the MDA content of PYH was significantly higher, indicating that the degree of membrane lipid peroxidation was more severe in PYH plants. The activity of SOD in roots and leaves of the two sesame cultivars significantly increased up to T2 and then decreased, while POD and CAT activities were increased along with the stress duration (**Figures 1B-D**, **Figures S3 B–D**). It is worth noting that the activity of antioxidant enzymes in JHM under drought stress conditions was significantly higher than in PYH, implying that JHM had a stronger enzymatic defense system than PYH. For instance, the activities of SOD, POD, and CAT in the leaf of JHM reached a maximum value of 166.10, 44.4, and 257.4 u/g FW (fresh weight), respectively, under the drought stress conditions compared to 119.5, 37.7, and 182.9 u/g FW, respectively, in PYH (**Figures 1B–D**).

**Figure 2:**
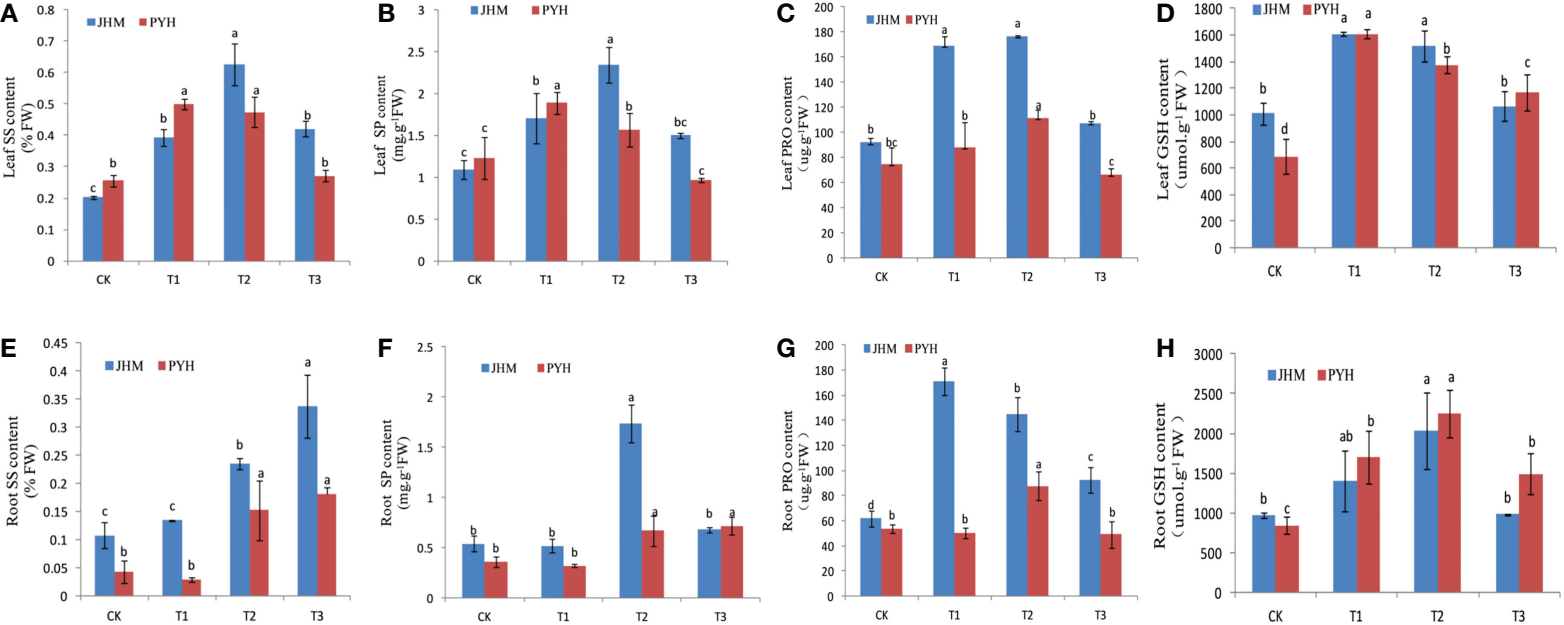
Variation in the content of osmolytes in JHM and PYH plants under drought stress conditions during anthesis. **(A–D)**. Leaf soluble sugar (SS), soluble protein (SP), free proline (PRO), and glutathione (GSH) contents, respectively. **(E–H)**. Their respective roots. CK, T1, T2, and T3 indicate plants were stressed for 0, 3, 5, and 7 days, respectively. The different lowercase letters indicate significant differences at the P<0.05 probability level.

We further assayed the content of osmolytes, including soluble sugar (SS), soluble protein (SP), free proline (PRO), and glutathione (GSH) in the leaf and root of the two sesame cultivars under the drought stress conditions at the different time points. Except for the roots’ SS content, both the osmolytes showed a significant increase of content in leaves and roots at T1 and T2 and then decreased at T3 (**Figure 2**), suggesting that prolonged drought stress of more than a week might severely affect sesame productivity. As expected, the increase in osmolytes contents in JHM was more considerable than in PYH (**Figure 2**).

### Transcriptome profiles of JHM and PYH plants under drought stress during anthesis

**Figure 3:**
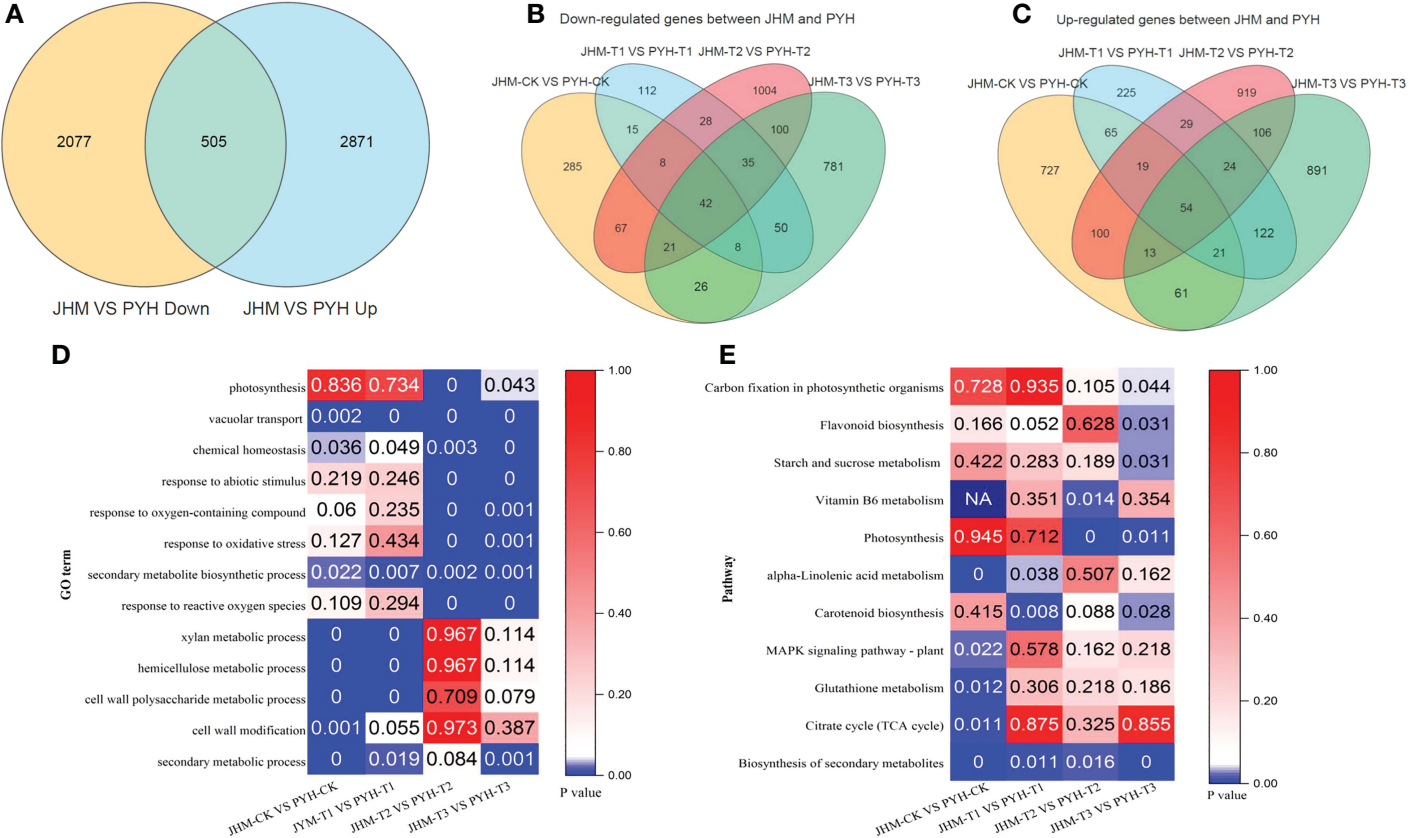
DEGs between JHM and PYH and their underlying metabolic processes. **(A)**. Venn diagram among DEGs between JHM and PYH. **(B)** and **(C)**. Venn diagram showing highly induced DEGs at all time points during the drought stress in JHM and PYH plants, respectively. **(D)**. Significant enriched GO terms of DEGs between JHM and PYH. **(E)**. Significant induced pathways that involve DEGs between JHM and PYH.

To get more insights into the drought-responsive mechanisms in JHM and PYH plants, roots samples of CK, T1, T2, and T3 from the two sesame cultivars in three biological replicates, were subjected to RNA sequencing *via* the Illumina sequencing platform. The summary of the transcriptomics data is presented in **Table S2**. The total clean reads generated varied from 38.01 to 55.45 million. The unique mapping reads matching the sesame reference genome ranged from 77.71 to 93.48% and 78.76 to 93.94% for JHM and PYH, respectively. Correlation analysis showed strong positive correlations between samples within the same group, indicating high reproducibility between the biological replicates (**Figures S4A**). We performed the principal component analysis (PCA) to differentiate between the groups. The results showed that the transcriptome of JHM and PYH roots was very different and changed according to the drought stress severity (**Figure 3**). There were 22674 genes (including 1142 novel genes) in all samples (**Table S3**). The total sequenced genes from all samples accounted for 94.45% of the sesame reference genome (**Table S3**). Reads alignment analysis showed that over 75% of the genes are located in exonic regions (**Figure S5**).

### Dynamic transcriptome changes in JHM and PYH plants along with drought stress severity during anthesis

**Figure 4:**
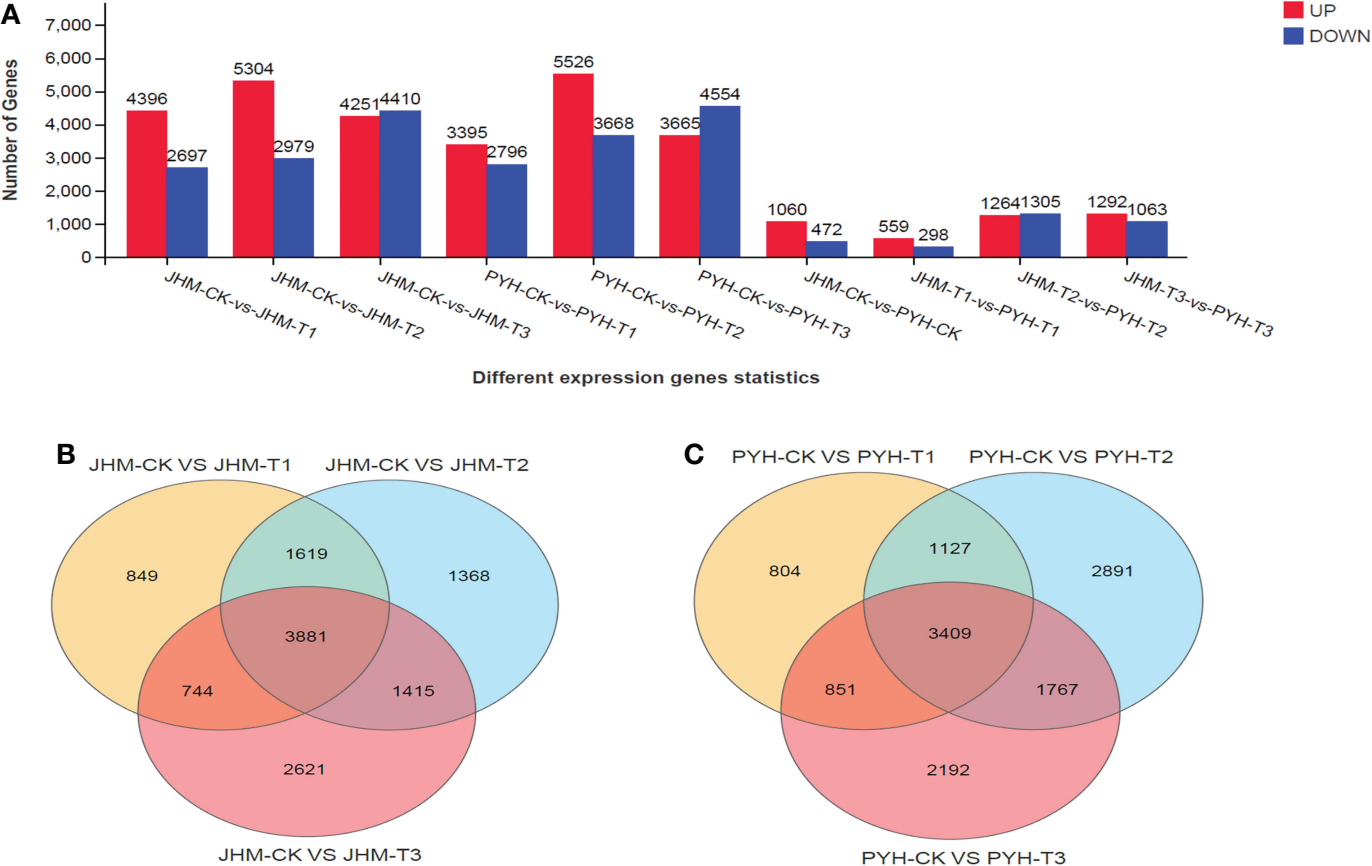
Differentially expressed genes (DEGs) along with the drought treatments in JHM and PYH plants. **(A)**. Number of up- and down-regulated genes at different time points in plants of the two cultivars. **(B, C)**. Venn diagram among DEGs at different time points in JHM and PYH, respectively.

To uncover drought-induced changes in transcriptional levels in the two cultivars during anthesis, we carried out differentially expressed genes (DEGs) analysis along with the drought duration. In total, we identified 24,037 DEGs, including 13,951 and 10,086 up- and down-regulated genes in JHM, respectively. Meanwhile, 23,604 DEGs, including 12,586 and 11,018 up- and down-regulated genes, respectively, were identified in PYH. In both cultivars, the number of up-regulated DEGs showed similar patterns of increasing and then decreasing from T2 (**Figure 4A**). In contrast, the number of down-regulated DEGs increased along with the drought stress duration. We searched for genes that were significantly affected at all time points in JHM and PYH. We detected 3,881 and 3,409 overlapped DEGs at the different time points in JHM and PYH, respectively (**Figures 4B, C**).

We performed GO and KEGG analyses to unveil molecular mechanisms involving the DEGs. The most GO terms that involve DEGs at early (T1) and moderate (T2) drought stress during anthesis in JHM included RNA modification, organic substance metabolic process, and ribonucleoprotein complex biogenesis (**Figure S6A**). At T3, the main GO terms that involve DEGs in JHM were hormone-mediated signaling pathway, regulation of meristem development, response to hormones, response to stimulus, signal transduction, and developmental growth. While in PYH, the most identified GO terms were stomatal movement, response to water deprivation, proteolysis, RNA modification, anion transport, and acetyl-CoA metabolic process at early and moderate drought stages, and response to water deprivation, ethylene metabolic process, anion transport, and steroid biosynthesis process at T3 (**Figure S6C**). Noteworthily, GO term related to the stomatal movement was identified only in PYH at the early drought stage and included four DEGs, ncbi_105159372 (PLDDELTA), ncbi_105162005 (AHK5), ncbi_105162425(CAS), and ncbi_105174436 (HSC-2).

The KEGG analysis revealed that several pathways related to plants’ drought stress tolerance mechanisms were significantly induced in JHM compared to PYH (**Figures S6B–D**). For instance, at T3, the DEGs in JHM were mainly assigned to photosynthesis - antenna proteins, biosynthesis of amino acids, fatty acid metabolism, peroxisome, lysine degradation, ascorbate and aldarate metabolism, pyruvate metabolism, plant hormone signal transduction, tryptophan metabolism, biosynthesis of secondary metabolites, and glutathione metabolism (**Figure S6B**). In contrast, in PYH, the most significant pathways at the same time were plant hormone signal transduction, MAPK signaling pathway, carbon metabolism, biosynthesis of amino acids, and arachidonic and alpha-linolenic acids metabolism (**Figure S6D**).

### DEGs between JHM and PYH plants and potential candidate genes for drought stress tolerance improvement in sesame

A total of 5,453 DEGs, including 505 common (up- or down-regulated), were identified between JHM and PYH (**Figures 4A**, **3A**). GO analysis revealed that at T2 and T3, most DEGs between JHM and PYH plants were related to vacuolar transport, chemical homeostasis, response to abiotic stimulus, response to oxidative stress, and secondary metabolites biosynthesis processes (**Figure 3D**). Meanwhile, the most induced pathways between JHM and PYH plants were photosynthesis, carotenoid biosynthesis, and biosynthesis of secondary metabolites (**Figure 3E**). Flavonoid biosynthesis and starch and sucrose metabolism were specifically significantly induced at T3. Interestingly, the DEGs enriched in photosynthesis were 2.0- to 7.9-fold more highly induced in JHM than in PYH.

In order to identify potential candidate genes for targeted improvement of drought stress tolerance in sesame, we constructed Venn diagrams among the up- and down-regulated DEGs between JHM and PYH. There were 54 and 42 DEGs significantly induced in JHM and PYH, respectively, at the four-time points (**Figures 3B, C**). It is worth noting that the expression of 11 up- and 11 down-regulated genes between JHM and PYH were highly affected (|FPKM| >10 during at least one-time point) along with the drought treatments. Thus, we selected these genes as potential candidate genes for future studies aiming to enhance drought stress tolerance in sesame (**Table S4**).

### Expression of glutathione and ethylene biosynthetic genes

**Figure 5:**
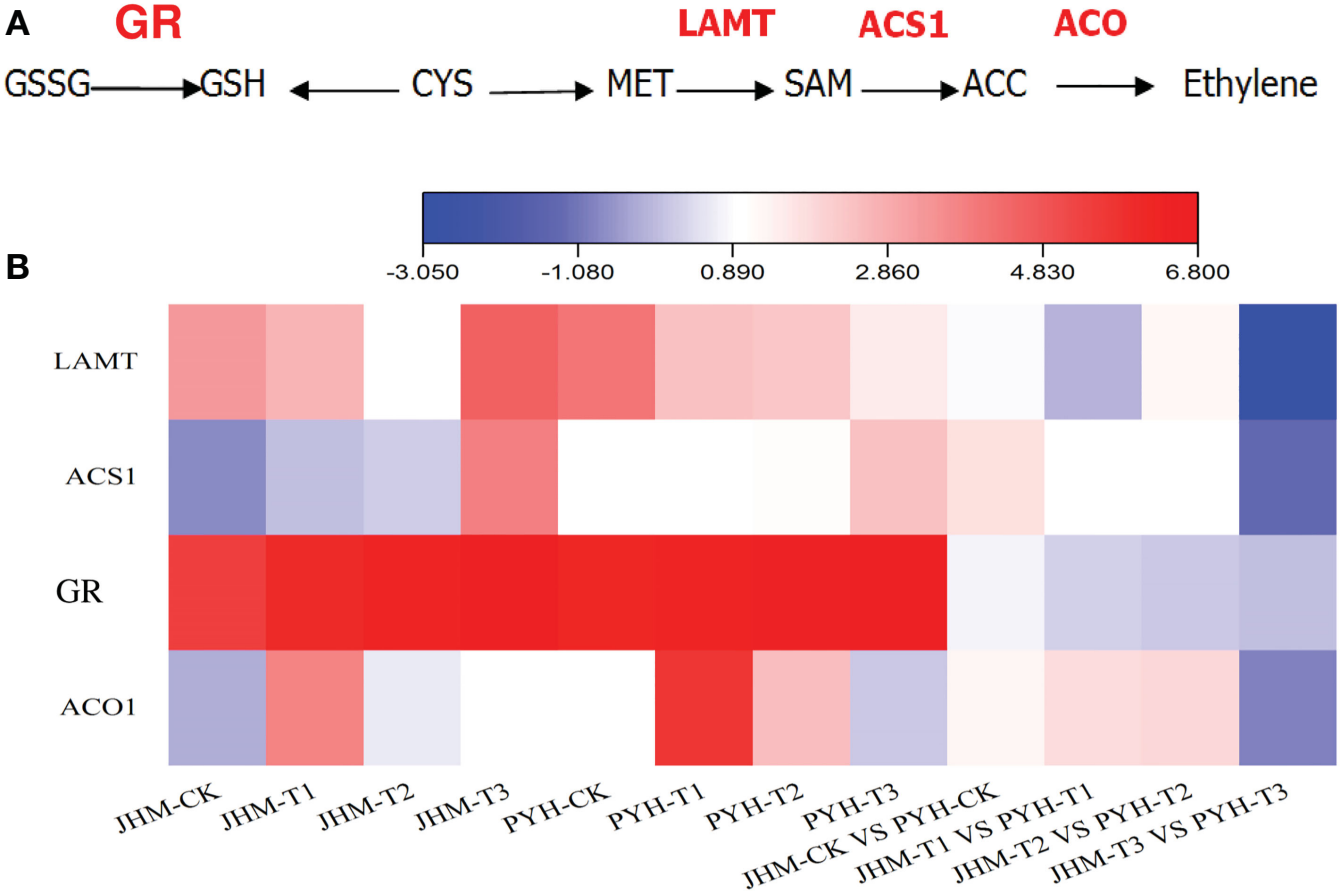
Expression patterns of glutathione and ethylene biosynthetic genes in JHM and PYH plants. **(A)** A diagram of glutathione and ethylene biosynthesis. **(B)** Expression patterns of glutathione reductase (GR) and ethylene biosynthetic genes in JHM and PYH plants under drought stress conditions.

Glutathione and ethylene play critical roles in plants’ tolerance to abiotic stresses. Glutathione reductase (GR) catalyzes to maintain cellular levels of reduced glutathione, which is essential for reactive oxygen species control (Couto et al., 2016). We then examined the expression of genes involved in the glutathione and ethylene biosynthesis pathway (**Figure 5A**), including At3g24170 (*GR*, glutathione reductase, ncbi_105158649), S-adenosylmethionine-dependent methyltransferase (*LAMT*, ncbi_105155206), 1-aminocyclopropane-1-carboxylate synthase (*ACS1*, ncbi_105164055), and 1-aminocyclopropane-1-carboxylate oxidase 1 (*ACO1*, ncbi_105161839). As shown in **Figure 5B**, the sesame *GR* was up-regulated along with the drought stress duration in both JHM and PYH plants. However, it was slightly more induced in JHM plants than in PYH plants. The expression of LAMT was also induced by the drought stress in the two cultivars. *ACS1* was mainly induced at T3, while *ACO1* at T1 (**Figure 5B**). At T3, the expression levels of the four genes were 1.0- to 8.1-fold highly induced in JHM plants than in PYH plants.

### Expression of transcription factors involved in drought stress regulation in JHM and PYH plants

**Figure 6:**
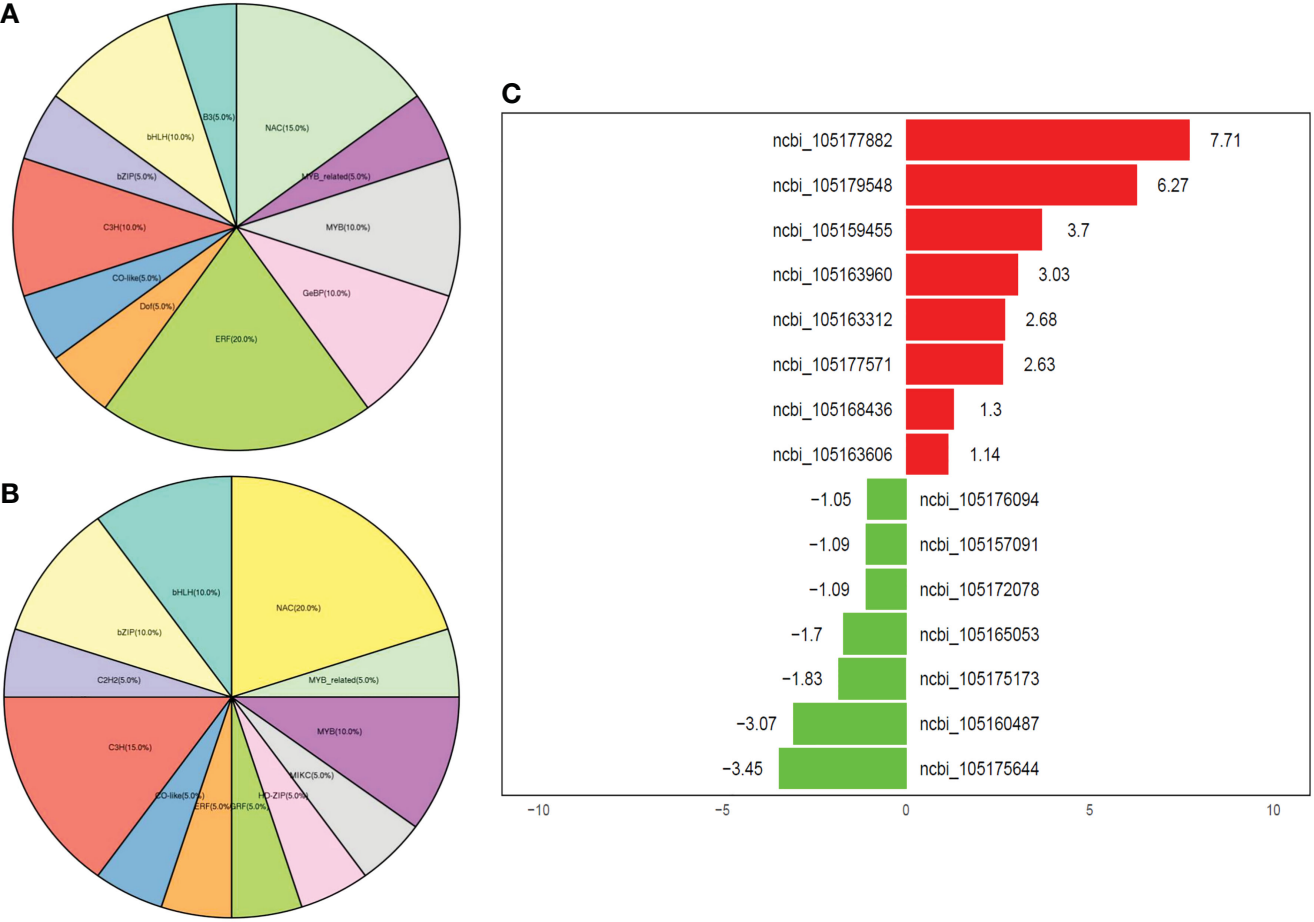
Overview of the expressed TF family genes in JHM and PYH plants under drought stress conditions. **(A, B)** Percentage of TF family genes that were expressed in JHM and PYH plants, respectively. **(C)** Top expressed TF family genes in JHM and PYH plants. Values represent the fold changes in gene expression. The red and the green color indicate up- and down-regulated genes, respectively.

We screened the expressed genes and found that the most expressed transcription factor families at all time points of the drought stress in JHM plants were ERF (20%), followed by NAC (15%), MYB (10%), GeBP (10%), C_3_H (10%), and bHLH (10%) (**Figure 6A**). Meanwhile, NAC (20%), C_3_H (15%), MYB (10%), bZIP (10%), and bHLH (10%) were the most expressed TFs in PYH plants (**Figure 6B**). We then filtered out the top expressed TFs in the two cultivars under the drought condition for future studies (**Figure 6C**). In JHM, the top expressed TFs included *ERF091* (ncbi_105175644), *LAF1* (ncbi_105160487), *JUB1* (ncbi_105175173), *NAC100* (ncbi_105157091), and *MYB4* (ncbi_105176094). These genes are known to be essential for plant survival from abiotic stresses. For example, *JUB1* is involved in various metabolic processes, such as trehalose, proline, hyperosmotic, and flavonoid biosynthetic processes (Alshareef et al., 2019; Chen et al., 2021).

### RT-qPCR validation

**Figure 7:**
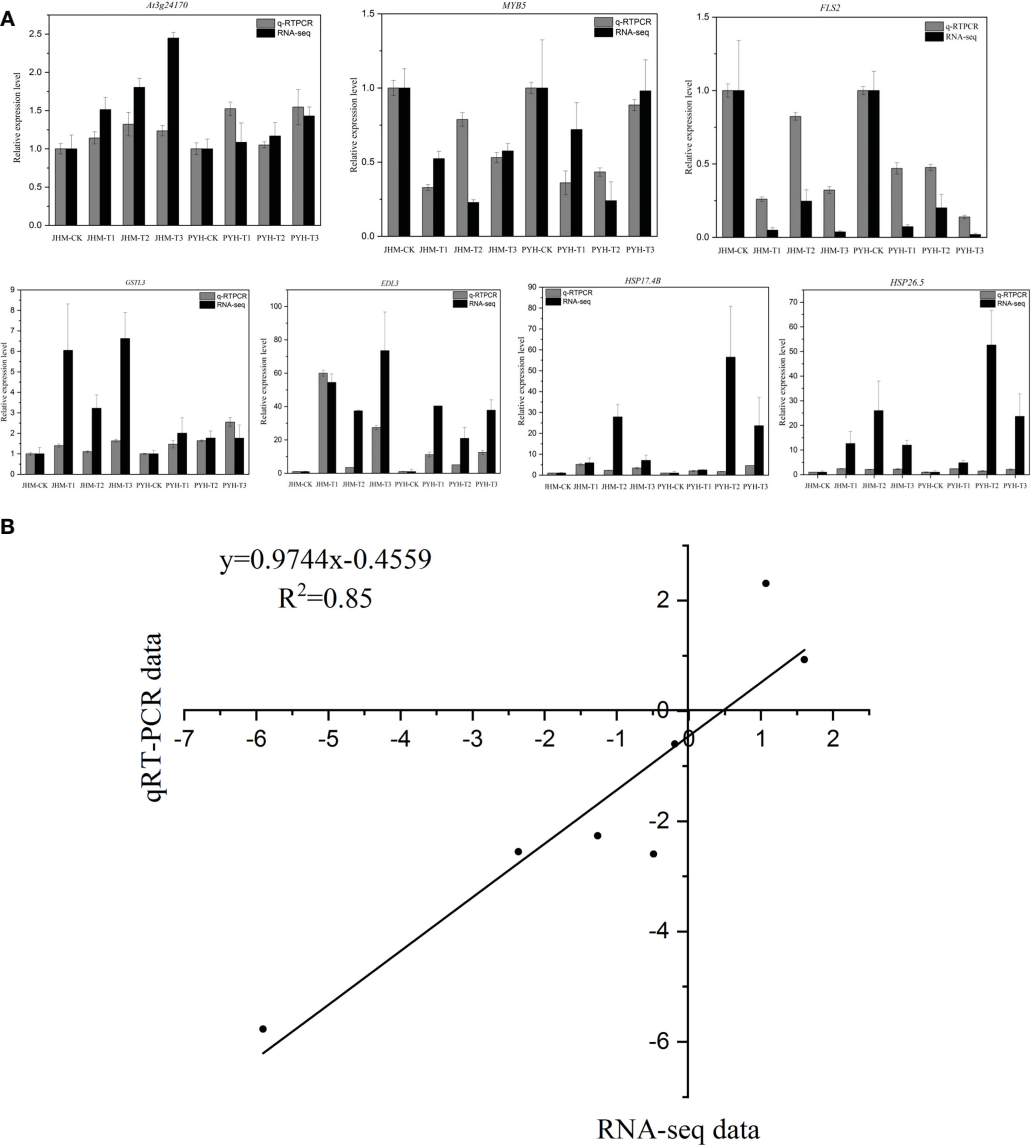
RT-qPCR validation of the expression levels of seven selected genes. **(A)** Expression patterns of each selected gene *via* RNA-seq and RT-qPCR. **(B)** Linear correlation analysis of RNA-seq and RT-qPCR data.

To validate the RNA-seq data, we selected seven genes for RT-qPCR analysis. As shown in **Figure 7**, the expression patterns of the selected genes *via* the RT-qPCR and RNA-seq were consistent (R^2^ = 0.85), confirming the reliability of our results.

## Discussion

Drought tolerance of crop plants involves complex and various physiological and molecular mechanisms that are not yet well understood (Schulz et al., 2020). In this study, we investigated the physiological and transcriptional responses of two black sesame cultivars to induced drought stress during anthesis. Although the drought tolerance capacity of plants of the two cultivars was very different, the drought-responsive mechanisms recorded were similar. The plants of both cultivars reacted to the induced drought by up- or down-regulating a set of genes, mainly by activating hormone and antioxidant-related genes. Accordingly, the activity of antioxidant enzymes POD, SOD, and CAT was significantly increased in both plants to curb the drought-induced accumulation of ROS. Under drought stress, electron transport chains in plant cells are devastated, giving rise to excessive ROS that causes oxidative stress, which in turn damages biological membranes and alters developmental processes (Labudda and Azam, 2014; Meng et al., 2014). Numerous other mechanisms, such as enhancement of the root system, reducing the stomatal aperture, and accumulation of different osmotic adjustment substances, are initiated by plants to cope with drought stress (Farooq et al., 2009; Kaur and Asthir, 2017). We observed an increase in the content of key osmoprotectants, including free proline, soluble sugar, soluble protein, and glutathione, in the roots and leaves of plants of the two cultivars under the induced drought stress. The accumulation of organic osmolytes in order to maintain cells’ homeostasis is a well-known mechanism by plants to resist drought stress (Bai et al., 2019). Similar mechanisms have been reported in sesame (Fazeli et al., 2007; Kadkhodaie et al., 2014; Dossa et al., 2017). Besides, it is demonstrated that plants initiate a series of TFs phosphorylation/dephosphorylation under stress to enable them to bind cis-elements of stress-related genes and enhance stress tolerance (Sardar-Ali et al., 2018; Baillo et al., 2019). TFs coordinate the expression levels of target genes to help plants maintain a particular phenotype (Feng et al., 2018). In sesame, previous studies mainly on white sesame revealed that ERF, MYB, bHLH, and WRKY family genes are the most induced by abiotic stresses (Dossa et al., 2019). Herein, we found that the most induced TFs in the two black sesame cultivars during the drought stress were ERF and NAC family genes. These results show that the different colored sesame response to drought is slightly different, and genotype may determine the ability of a sesame cultivar to resist or be susceptible to drought stress.

Compared to PYH, JHM plants showed higher tolerance to the induced drought stress. As shown in the GO analysis results, plants of PYH closed their stomata at the early drought stage, while plants of JHM maintained their photosynthetic activities up to the severe drought stage. Closing of stomata under water deficit suppresses transpiration and blocks CO_2_ exchanges, leading to a reduction of photosynthesis and alteration of developmental processes (Ullah et al., 2022). Through the analysis of JHM plants’ adaptation mechanisms to the induced drought stress, it could be inferred that stimulating a high number of genes, especially genes involved in ROS scavenging, hormone, and glutathione-related genes, and promoting the accumulation of organic osmolytes might be efficient strategies to improve black sesame drought tolerance. Osmoprotectants with high lipophilicity increase the turgor pressure and trigger water uptake from soil under abiotic stress (SeyedYahya and Hamideh, 2016; Blum, 2017). Furthermore, glutathione metabolism and ethylene formation are essential for plants’ resistance to abiotic stress (Bhargava and Sawant, 2012; Huang et al., 2019; Khan et al., 2015). In wheat, the up-regulation of ethylene biosynthetic genes *ACO*, *ACS1*, and *ACS2* conferred better tolerance to water deficit (Luo et al., 2021).

The integration of results from the physiological and transcriptomic analyses highlights the importance of a strong antioxidant system, osmoprotectants’ biosynthesis and accumulation, TFs, and phytohormones (ethylene biosynthetic pathway) for drought tolerance in black sesame. Key genes in these pathways might be investigated to deepen our understanding of black sesame drought response and provide resources for sesame improvement. Although the JHM exhibited higher tolerance to drought, plants of the two black sesame cultivars showed a decrease in the number of expressed genes, antioxidative processes, and the content of osmolytes after seven days of drought treatment during anthesis. These results indicate that progressive or prolonged drought of more than a week may significantly affect black sesame productivity and quality. In addition, they show that more interest should be given to sesame plants’ drought tolerance improvement. Numerous candidate genes for drought and other abiotic stress tolerance in sesame have been identified (Dossa et al., 2020; Dossa et al., 2021; Su et al., 2022). However, functional genomics studies are lacking to enable the molecular breeding of drought-tolerant sesame varieties. In this study, we selected 31 potential candidate genes for black sesame drought resistance improvement. They included five TF family genes (*ERF091*, *LAF1*, *JUB1*, *NAC100*, and *MYB4*) with the highest FPKM values in the JHM plants. These genes might govern the higher tolerance capability of JHM plants to the induced drought stress. Therefore, great efforts are then to be made to functionally characterize these genes and dissect the regulatory networks of drought response in sesame.

## Conclusion

In summary, this study provides insights into the drought-responsive mechanisms of black sesame during anthesis by assessing dynamic physiological and transcriptional changes in two sesame cultivars (JHM and PYH). Compared to PYH, JHM plants exhibited high tolerance to the induced drought stress. We found that increased tolerance to drought stress in black sesame is associated with significant induction of key pathways and genes involved in plants’ abiotic stress response. Principally, genes that are involved in hormone signaling processes, glutathione and ethylene biosynthesis, photosynthesis, reactive oxygen species metabolic processes, biosynthesis of osmoprotectants, and secondary metabolites biosynthesis. These genes mediated efficient osmotic adjustment, ROS scavenging, and maintenance of biological membranes’ stability and cellular processes in JHM plants under induced drought stress during anthesis. Both the drought-responsive mechanisms decreased in plants of the two cultivars after seven days of drought treatments, indicating that prolonged drought stress of more than a week might severely affect sesame production. We identified 31 potential candidate genes for drought stress tolerance improvement in black sesame. Our results represent fundamental resources for further studies towards the dissection of the regulatory networks of drought stress response in sesame and molecular breeding of drought-tolerant varieties.

## Data availability statement

The datasets presented in this study can be found in online repositories. The names of the repository/repositories and accession number(s) can be here: https://db.cngb.org/search/project/CNP0003731/.

## Author contributions

XW, GY, and ZWu conceived and designed the experiment. XW, HY, GW, TS, ZWa, and MW helped in conducting the experiment. SF helped in the gene expression experiment. XW statistically analyzed the data and wrote the draft. SF and ZWu helped in revise the final manuscript.
