# Supplementary material for: Comparative analysis of drought stress-induced physiological and transcriptional changes of two black sesame cultivars during anthesis
Source: Front Plant Sci. 2023 Feb 21;14:1117507. doi: 10.3389/fpls.2023.1117507 (PMC9989188; doi:10.3389/fpls.2023.1117507)
Supplement: Supplementary file 1 [file Presentation_1.pdf]

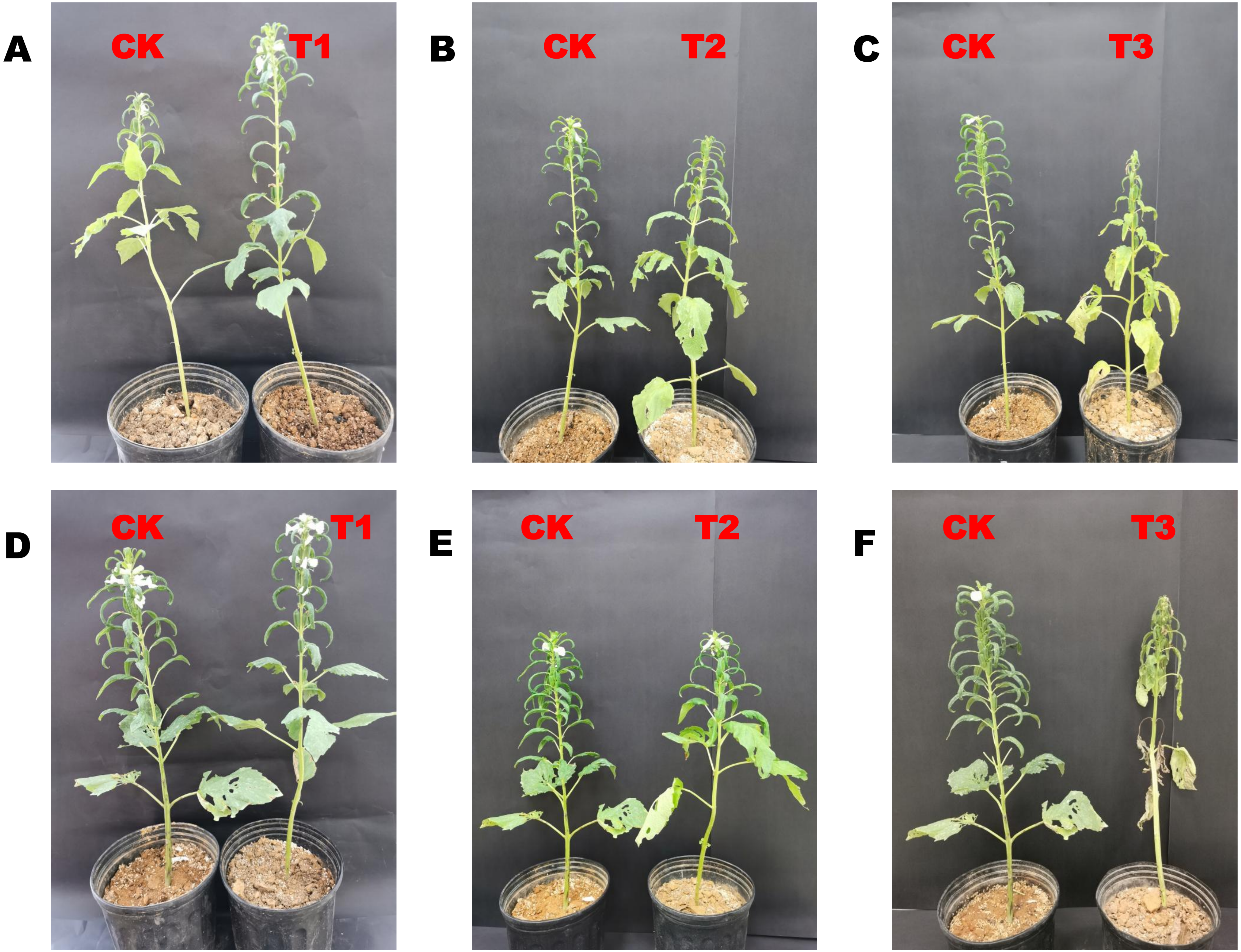

Fig S1. Changes in morphological characteristics in response to drought stress of JHM (A-C) and PYH (D-E). CK, T1, T2, and T3 indicate plant stressed for 0, 3, 5, and 7 days, respectively.

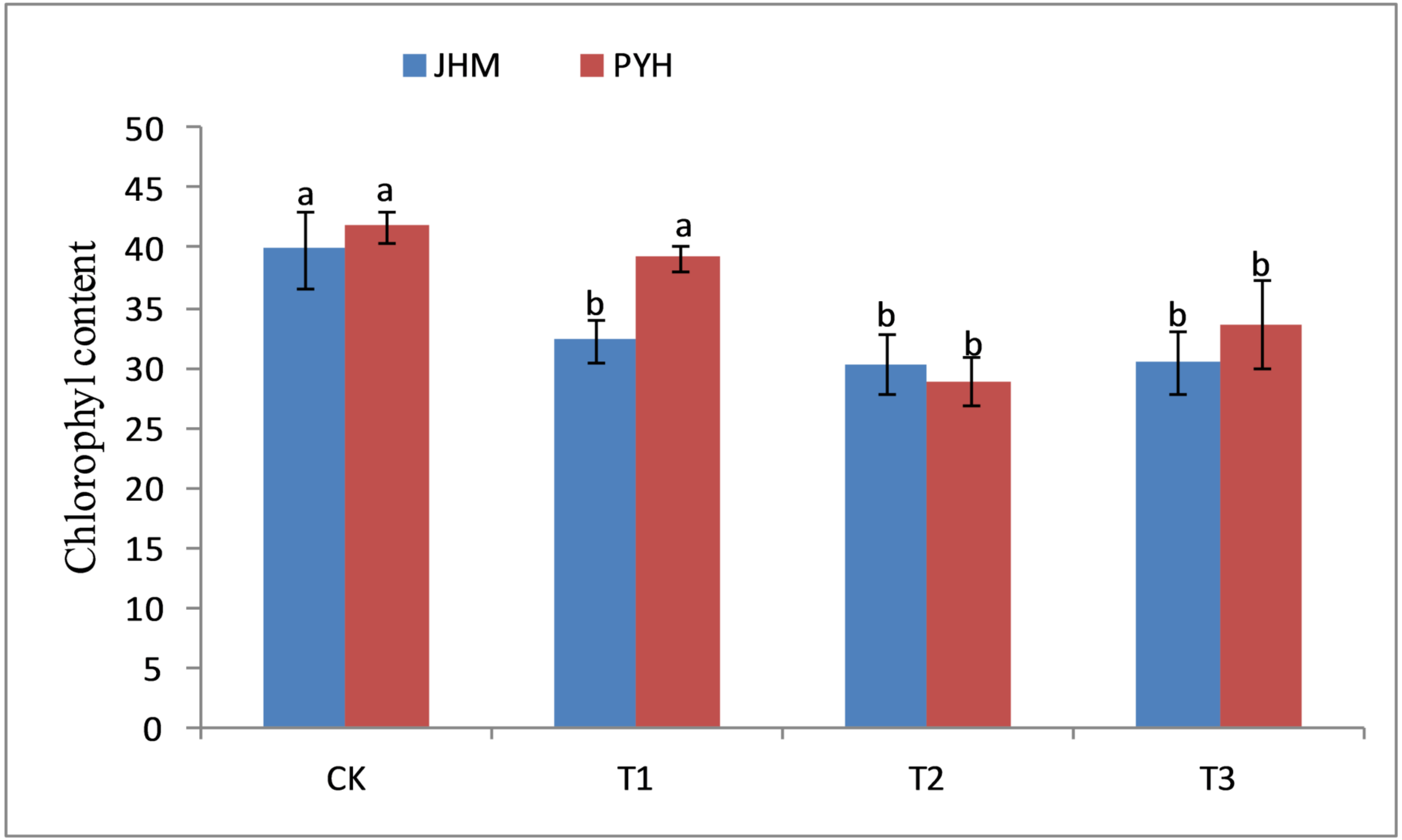

Fig S2. Chlorophyll content of JHM and PYH under drought stress during anthesis. CK, T1, T2, and T3 indicate plant stressed for 0, 3, 5, and 7 days, respectively.

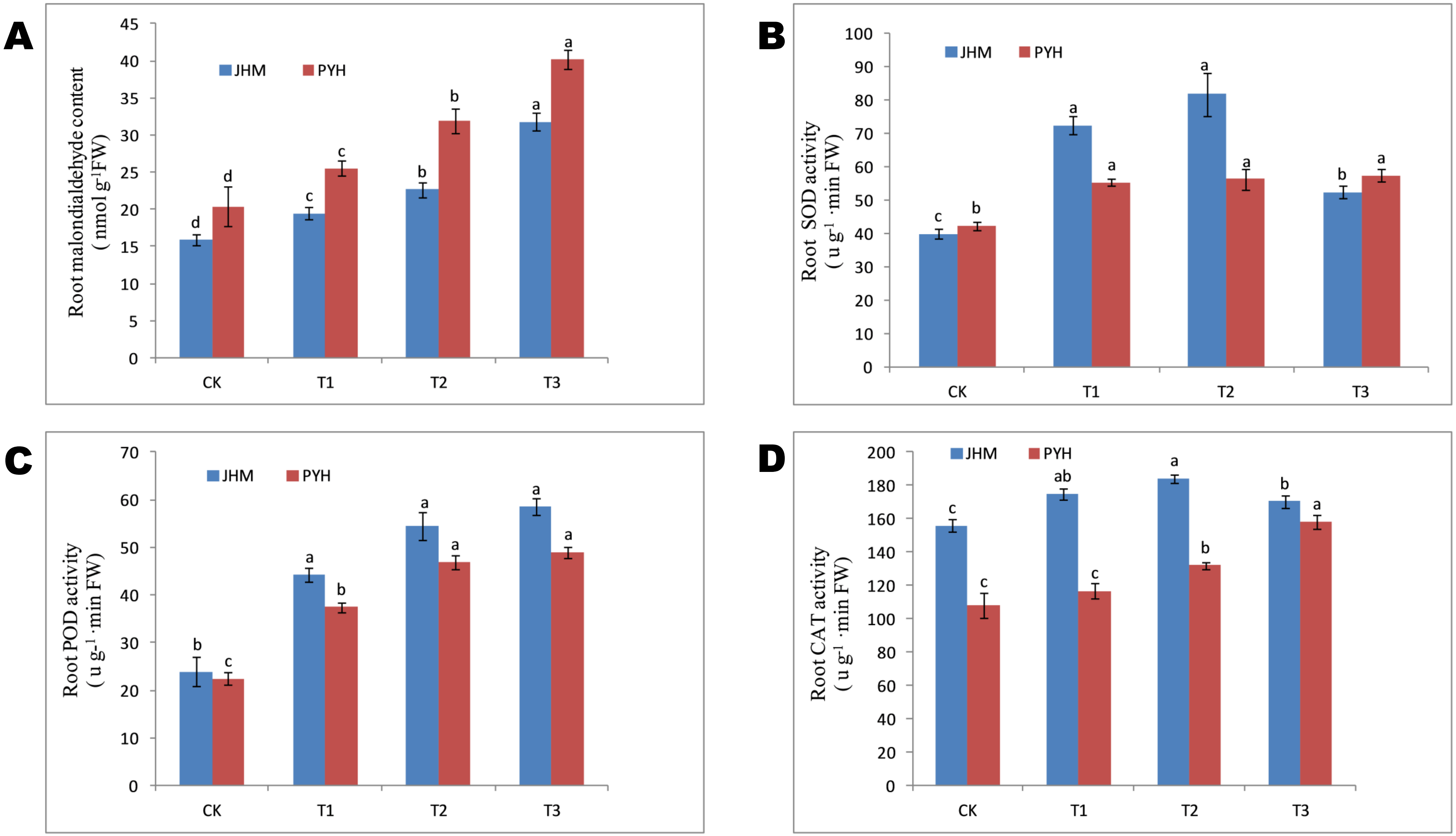

Fig S3. Antioxidation status in roots of JHM and PYH under drought stress during anthesis. A. Malondialdehyde content; B. Superoxide dismutase activity; C. Peroxidase activity; D. Catalase activity. CK, T1, T2, and T3 indicate plant stressed for 0, 3, 5, and 7 days, respectively.

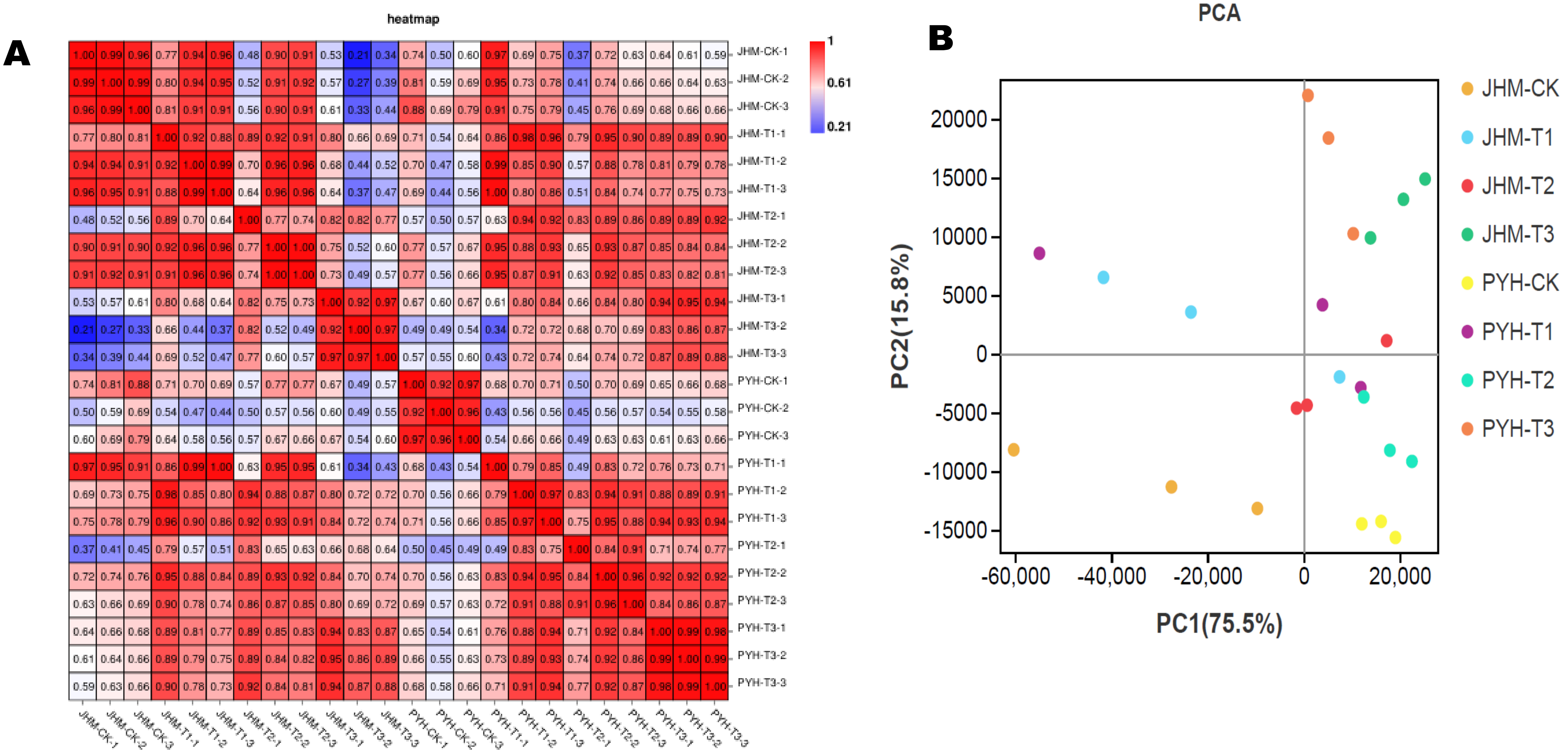

Fig S4. Correlations analysis (A) and Principal component analysis, PCA (B) of JHM and PYH root samples' transcriptome under control and drought stress conditions at different time points.

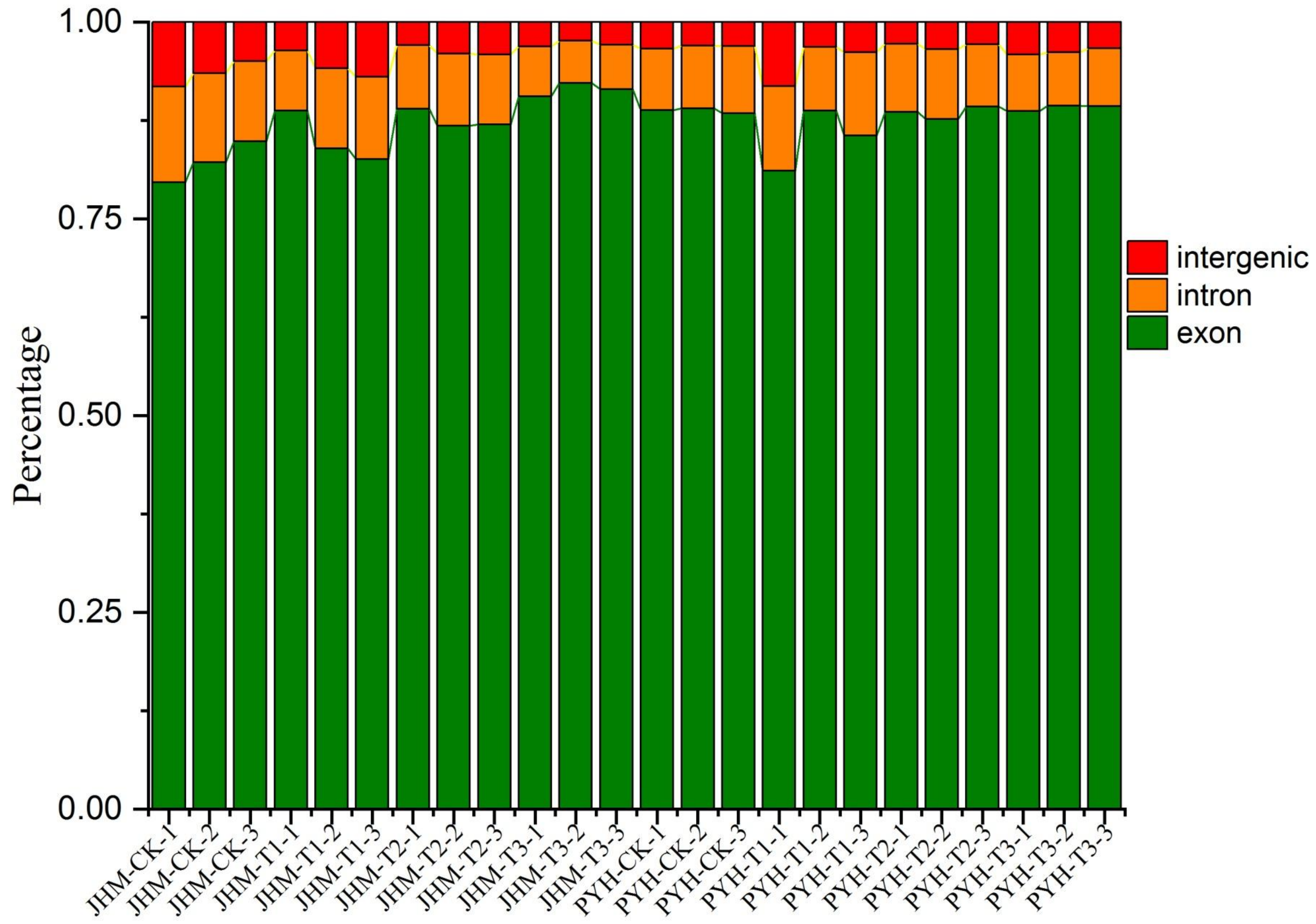

Fig S5. Reads alignment results of genes identified in JHM and PYH root samples.

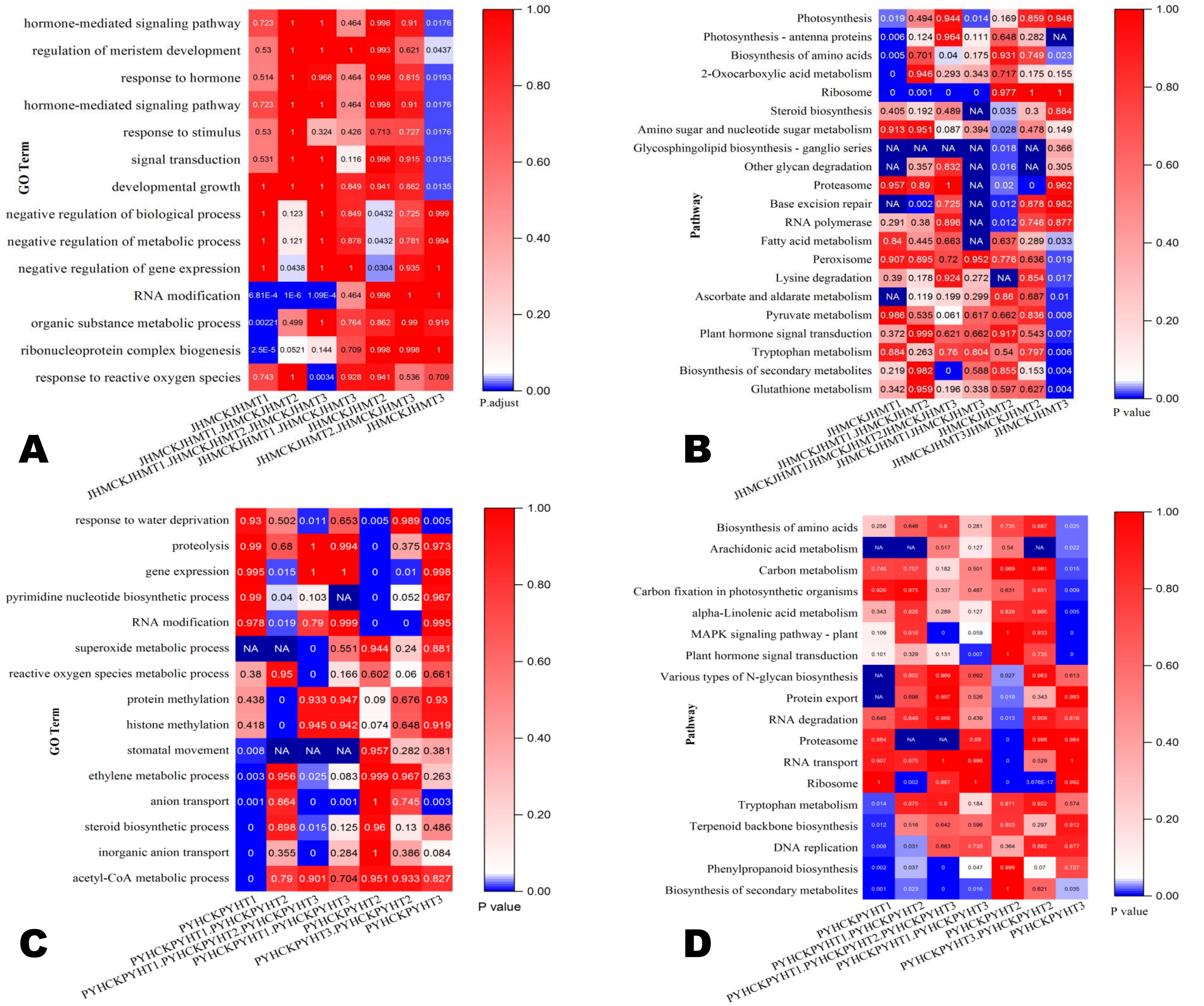

Fig S6. GO and KEGG analysis results of DEGs identified in JHM and PYH at different time points of drought stress during anthesis.

A and C, GO results of DEGs in JHM and PYH, respectively.

B and D, KEGG results of DEGs in JHM and PYH, respectively.

Note: The significance of the most represented terms is indicated by a *p*-value. Significant *p*-values are indicated in blue, whereas non-significant *p*-values are indicated in red.
